# Relationship between Weight Status and Health-Related Quality of Life in a Sample of Early Adolescents from Central and Northern Italy: A Cross-Sectional Study of the AVATAR Project Participants

**DOI:** 10.3390/ijerph18168782

**Published:** 2021-08-20

**Authors:** Francesca Mastorci, Paolo Piaggi, Cristina Doveri, Gabriele Trivellini, Irene Marinaro, Anselmo Casu, Alessandro Pingitore, Cristina Vassalle

**Affiliations:** 1 Clinical Physiology Institute, CNR, 56121 Pisa, Italy; mastorcif@ifc.cnr.it (F.M.); cristina.doveri@ifc.cnr.it (C.D.); gabriele.trivellini@ifc.cnr.it (G.T.); irene.marinaro@ifc.cnr.it (I.M.); webmaster@easywebmaster.eu (A.C.); 2 Department of Information Engineering, University of Pisa, 56121 Pisa, Italy; paolo.piaggi@gmail.com; 3 Fondazione G. Monasterio, Regione Toscana, 56121 Pisa, Italy; cristina.vassalle@ftgm.it

**Keywords:** adolescence, health-related quality of life, overweight, obesity, underweight, sex differences

## Abstract

Among the various factors that could influence health-related quality of life (HRQoL) in adolescence, body mass index (BMI) seems to play a key role as a main anthropometric parameter. The aim of this cross-sectional study was to determine, in a sample of Italian adolescents, whether HRQoL is associated with the different weight status categories (underweight, normal weight, overweight, obese), according to BMI cut-off points for children, even considering sex differences. Data were collected from 1707 adolescents (*n* = 828 males) in seven schools. HRQoL was analyzed using the Italian version of KIDSCREEN-52. Males were more overweight and obese than females (13% vs. 10% and 4% vs. 2%, *p* < 0.05, respectively). In females, BMI categories are associated with physical well-being (*p* < 0.05), emotion/mood (*p* < 0.05), self-perception (*p* < 0.001), financial resources (*p* < 0.05), and bullying behavior (*p* < 0.05). In males, weight status is linked to physical well-being dimension (*p* < 0.001) and perception of self (*p* < 0.05). Our results may suggest that there is an association between weight status categories and HRQoL, more pronounced in females than in males. Interestingly, the weight status correlated more with the psychological dimension mainly in females, whereas in males, a stronger association between weight and physical status was observed, suggesting that given the complex, multifaceted, and dynamic nature of relationship between health-related quality of life and weight status in adolescents, multiple factors must be considered.

## 1. Introduction

Adolescence is a period characterized by important changes in growth and physiological and behavioral development, associated with emotional, social, and cognitive alterations [1]. Many adolescents experience the transition to adulthood by enhancing resilience, while others report a psychosocial vulnerability, which materializes in a compromise in relationships with parents and peers and a decrease in self-esteem, resulting in decreased health-related quality of life (HRQoL) [2]. Among the various factors that could contribute to reduced well-being perception, body weight is considered as a main anthropometric parameter, and both underweight and overweight (up to severe obesity) have an important psychosocial impact [3,4]. This is especially relevant when we consider that obesity and overweight in children and adolescents are recognized as a major public health problem worldwide. In recent years, an increase in Europe has been observed, especially in the Mediterranean area, due to a low adherence to a Mediterranean diet [5,6,7].

Regarding HRQoL, the relationship between obesity and self-esteem has often been evaluated from the psychosocial point of view, revealing a strong association between low perceived levels of well-being and quality of life. However, a few studies described the association between other weight categories (such as underweight) and well-being [8]. In Griffiths’ systematic review, the authors highlight a relationship between self-esteem and quality of life in obese children and adolescents, while the subthreshold psychological morbidities associated with weight change are not well established [8]. In the study of Pu et al., reduced health perception and underweight were associated in a population of adolescents from Taiwan [4]. However, whether or not the close association between well-being, body weight, and sex differences is evident in this context, the relationship between the single components of well-being, considered in terms of physical, emotional, and social perspectives, and body weight is less clear. Moreover, it is also noteworthy that perception of own well-being in relation to body weight may have also cultural implications, as Viner’s study has shown [9]. Therefore, taking into account these points, this cross-sectional study, included in the main AVATAR project, was focused on assessing the potential association between weight status, as assessed through the body mass index (BMI), and the different dimensions of HRQoL, even considering sex differences, in a sample of healthy early adolescent students, in order to help with the definition of preventive strategies.

## 2. Materials and Methods

### 2.1. Participants and Procedures

Data in the AVATAR study were collected between 2017 and 2018 by means of a web platform. Seven junior high schools were enrolled in the AVATAR (“A new purpose for promotion and eVAluation of healTh and well-being Among healthy teenageRs”) project on a voluntary basis [10,11,12]. Participating schools are located in North Italy, mainly in Tuscany, one in Liguria, and two in Friuli Venezia Giulia. In total, 1932 students were included. Adolescent students were enrolled according to the following inclusion criteria: age 10–14 years (early adolescence), absence of neuropsychiatric or other diseases, informed consent signed, and filling of the entire questionnaires proposed. Among them, exclusion regarded 37 students for diagnosed neuropsychiatric or other diseases, 45 who did not sign informed consent, and 143 who did not complete all the questionnaires. Therefore, the final population consisted of 1707 adolescents.

Participants were previously instructed on how to fill out the questionnaires and how to conduct the tests. One or two project members visited each school to provide the adolescents with verbal and written information about the data collection. Written information was also distributed to the parents. Active informed consent was obtained from both adolescents and their parents or legal guardians.

All tests were conducted during the participants’ computer lesson in school time. No incentive was provided to adolescents or parents. A research assistant was available to provide information and technical support to complete questionnaires.

### 2.2. Ethics

All procedures performed in the study were in accordance with the ethical standards of the institutional and/or national research committee and with the 1964 Helsinki declaration and its later amendments or comparable ethical standards. The AVATAR project has been accepted by the Regional Pediatric Ethics Committee (Azienda Ospedaliero Universitaria Meyer) (code 166/2018).

### 2.3. Measures

Weight and height were measured by trained field workers according to WHO age group [10,11,12,13].

Body mass index (BMI) was calculated as weight (kg)/height^2^ (m^2^). Adolescents were classified into four weight status categories: underweight, normal weight, overweight, and obesity according to BMI cut-off points for children, endorsed by the International Obesity Task Force (IOTF) [14].

The Italian version of KIDSCREEN-52 was used to assess health-related quality of life [15,16]. The KIDSCREEN is a self-report questionnaire designed to address health-related quality of life, aimed to monitor and measure the personal experiences in children and adolescents concerning their perception of health status and well-being. The questionnaire, which describes physical, psychological, mental, social, and functional aspects of well-being, consists of 52 items grouped in 10 dimensions (Physical well-being, Psychological well-being, Moods and emotions, Self-perception, Autonomy, Parent relations and home life, Social support and peers, School environment, Social acceptance (bullying), Financial resources) [15,16]. Some sample items include “In general, how would you say your health is?” for Physical well-being dimension; “Have you felt satisfied with your life?” for Moods and emotions; “Have you been happy with the way you are?” for Self-perception. Cronbach’s alphas range from 0.77 to 0.89 for the dimensions of the 52-item version. Except for mood and bullying, higher values of the variables express a better health-related quality of life. KIDSCREEN questionnaires are psychometrically tested using data obtained in a multicenter European study which included a sample of 22,827 children recruited in 13 countries [17].

### 2.4. Statistical Analysis

Statistical data analyses were performed using StatView software. Data are presented as mean ± SD. Categorical variables were presented as counts and percentages. The Shapiro–Wilk test was used to assess the normality of data distribution. Two-way analysis of variance (ANOVA) was adopted to explore differences in KIDSCREEN-52 dimension scores across weight status categories and sex. Changes in BMI and health-related quality of life by sex were analyzed by Student’s unpaired *t*-test. Two-way ANOVA was used to assess differences in BMI categories according to KIDSCREEN-52 dimensions in the two sexes, and Scheffe test for post hoc analysis. Sensitivity analyses conducted after merging overweight subjects with subjects with obesity led to similar results. A *p* value ≤ 0.05 was considered statistically significant.

## 3. Results

### 3.1. Socio-Demographic Characteristics of Study Population

Table 1 shows the sample demographic characteristics and BMI categories. In total, 1707 participants (51% girls) were included in the analyses. There were no statistically significant differences between sexes concerning age and BMI value, calculated as weight (kg)/height^2^ (m^2^).

When we considered the weight status in the different categories, expressed as percentage of boys and girls in under and normal weight, overweight, or obesity (Table 1), boys turned out to be more overweight and obese, respectively (*n* = 105, 13% and *n* = 33, 4%, versus *n* = 89, 10% and *n* = 18, 2% in females, *p* < 0.05).

### 3.2. Sex Differences on Health-Related Quality of Life

Descriptive data of health-related quality of life in our sample divided by sex are presented in Table 2. All variables were normally distributed according to the Shapiro–Wilk test. Regarding HRQoL, several variables significantly differed according to sex. Males perceived a higher physical well-being (*p* < 0.001), emotional background (*p* < 0.05), perception of self (*p* < 0.01), and autonomy (*p* < 0.001) as compared to the female population, which instead reported a better awareness of financial resources (*p* < 0.001). There was also a sex difference regarding social context, in particular in the school environment, where females reported significantly higher levels than males (*p* < 0.001).

### 3.3. Relationship between Weight Status Categories and Health-Related Quality of Life in Female and Male Cohorts

Results of the primary analysis of health-related quality of life divided by sex in function of weight status categories are shown in Table 3. In the female population, to belong to one of the four categories of BMI influences the perception of physical well-being (*p* < 0.05); in particular, among females, underweight girls showed a higher score in this dimension than obese girls (*p* < 0.05). Additionally, BMI categories are associated with emotion/mood area (*p* < 0.05); post hoc analysis reveals that underweight girls presented a higher mood as compared to their obese counterparts (*p* < 0.05). BMI classification correlated with self-perception dimension (*p* < 0.001); in detail, normal weight girls revealed a better self-perception than overweight (*p* < 0.01) and obese girls (*p* < 0.05), and underweight females had a higher perception of themselves, understood as bodily perception, as compared to their overweight (*p* < 0.01) and obesity counterparts (*p* < 0.01). Moreover, belonging to the different categories of BMI is linked with the perception of financial resources (*p* < 0.05) and bullying behavior (*p* < 0.05). In the male population, the physical well-being dimension was associated with weight status (*p* < 0.001); particularly, normal weight boys had a higher perception of their physical health as compared both overweight (*p* < 0.05) and obese boys (*p* < 0.05). Furthermore, BMI categories are correlated with males’ perception of self (*p* < 0.05); post hoc analysis showed that obese boys had a lower body perception than their normal weight (*p* < 0.05) and underweight (*p* < 0.05) counterparts.

Exploring the impact of sex and BMI categories on levels of HRQoL, only the self-perception dimension decreases with the growth in BMI (obesity category), but more in females than in males (*p* < 0.05).

## 4. Discussion

The starting point of this cross-sectional study conducted on a sample of early adolescent students was that Italian girl adolescents, considering the single variables of HRQoL, had lower scores in psychological dimensions, in line with previous evidence obtained in a European sample, in which boys reported higher physical appearance, self-esteem, and mood dimensions [11,12,18,19,20,21,22]. When we considered HRQoL as a function of the different BMI categories, we observed that in females, this relationship was much closer than that found in males.

Thus, the main results of this study can be summarized in the following points: (i) in our female population, physical well-being, mood, and self-perception dimensions correlated with weight status, showing that underweight girls exhibited a higher score in these sub-scales as compared to their overweight and obese counterparts; (ii) in our male cohorts, BMI categories were associated only with physical well-being and self-perception, which were higher in normal weight than obese boys.

This study showed that in our sample, there are relevant differences in the association between HRQoL and body weight status, in particular regarding psychological variables. However, the results in this field are conflicting, probably due to heterogeneous target populations, clinical and healthy, male and female, and linked to other different factors such as cultural and country-specific ideology about weight status role; moreover, several studies have focused on the relationship between weight status and HRQoL in children, rather than adolescent samples. In this regard, our population involved adolescent students at Italian junior high schools, with a strict range of age of 10–14 years.

In addition to the data confirming that girls have a lower well-being perception, only underweight females, and not underweight boys, had significantly higher physical well-being and emotional functioning as compared to the obese group. This dichotomy is in line with previous studies, where adolescent girls exhibited higher levels of depressed mood and anxiety than boys [23,24]. Our findings are in line with the evidence showing that thinness was more common in girls than in boys [25]. Unlike our results which showed no associations with the social context, other evidence indicates that underweight adolescents are likely to also have problems in social relationships, both with friends and with family [25].

Our results could also be interpreted in relation to the Western sociocultural context in which adherence to the thinness model is a factor of body satisfaction. Although in our study the context of origin was not considered, they were all adolescents residing in Italy. In this regard, Viner et al. have shown that the association between psychological dimension, such as self-esteem and stress perception, and BMI depended on ethnic cultural traits [4,9]. For example, US Black adolescents report a less negative influence on the psychological domain of obesity compared with white adolescent females [26].

However, in general, this prototype of body satisfaction in females, although linked to the social and cultural context, could, in the long term, be associated with emotional distress, psychiatric disturbances, depression, and eating disorders including dieting and binge eating [27]. In general, adolescence, both for girls and boys, represents a critical period for body image development due to psychosocial transition. Despite this, our results, although acquired in a sample of early adolescents and thus not representative of adolescence in toto, are in line with the notion that suggests that body satisfaction differs between adolescent girls and boys; in fact, thinness represented the ideal body image for girls, and this is probably responsible for a better HRQoL score in thin girls. In a meta-analysis, Grosz et al. have found a reduction in body satisfaction in girls under the age of 19 due to the beauty image of the media, pointing out how beauty trends displayed in television or in social media can contribute to unhealthy adolescent body perceptions [28]. In industrialized countries, self-acceptance is considered an important psychological construct, predictive of body satisfaction in adolescents or, on the contrary, of body dissatisfaction, associated with unhealthy lifestyle habits, increased distress, and poor health [29]. Our data showing low self-esteem and high depressive mood in overweight and obese girls suggested how this condition can alter the perception of well-being, up to impaired HRQoL, leading, in the long term, to stigmatization, social marginalization, and higher incidence of diseases in adulthood.

The correlation between HRQoL and weight excess appears to differ between sexes [30]. In fact, it is remarkable that only in girls, during early adolescence, the physical component was stronger in the relationship with BMI status, probably in line with previous evidence that pointed out the natural tendency of boys to value corporeal well-being [29]. Our findings are in accordance with the previous observation suggesting an effect of sex in moderating the association between weight status and HRQoL. In particular, our results are consistent with other studies that reported that obese girls exhibited lower score in HRQoL compared to their normal weight counterparts, whereas no differences were described in obese boys. Furthermore, among girls, HRQoL was higher for thin girls and decreased with increasing BMI [31]. Although little is known about the factors associated with reduced HRQoL among overweight or obese adolescents, it is likely that increasing weight status has a moderate to strong negative influence on HRQoL in youth populations. Longitudinal studies suggest that obese females have a decrease HRQoL, above all in self-esteem score, not seen in males [32]. Conversely, Wake et al. found that overweight or obese boys may be more at risk of lower HRQoL than girls, demonstrating a fragmentation of results in the association between HRQoL and sex [33].

It is important to keep in mind that our sample is not representative of the country and affects a particular time window during adolescence, that is, “early adolescence”. This phase, from a psychological point of view, is characterized by low levels of future orientation, low risk perception, but an increase in risk taking behavior.

In terms of association between body weight status and social contexts, the findings of the current study indicated that no significant links, in the different settings (school, family, peers), existed with BMI categories for our population. This result is in part in line with a previous study that had asserted that the relationship between overweight and obesity status and poor HRQoL is not affected by socioeconomic position [34]. However, as concerns social acceptance, underweight and normal weight females were less likely to be bullied, whereas overweight and obese girls were more likely to be bullied. Being overweight and/or obese is one of main cause for youth bullying, with subsequent greater likelihood of developing sleeping problems, headaches, anxiety, low self-esteem, depression, and substance abuse [35,36]. The strength of this study is that little is known about the relationship between HRQoL and body weight categories in Italian teenagers despite the increase in Italy and other industrialized countries of overweight or obesity, probably due to a reduction in the Mediterranean nutritional lifestyle [37]. Our results, although not representative of all of Italy, suggest the importance of extending the survey to all Italian regions, thus building a representative group in order to help with the definition of preventive strategies.

The investigation of the relationship between weight status and each component of well-being is consistent with the evidence that body weight is a potential predictor of health-related quality of life in adolescents and thus may help to develop targeted preventive strategies. However, the calculation of BMI represents a limitation because it does not distinguish between lean and fat mass and does not identify the type of obesity. In addition, the use of self-reports for psychological evaluation could also be a limitation. Moreover, an important limitation is the study group that consisted of adolescents only 10–14 years old, thus during early adolescence, which cannot be considered representative of all adolescence; in addition, the schools involved in the AVATAR project, at the moment, are located in North Italy. In a culturally very diverse country, such as Italy, the results could have been very different with schools from different territories. Another important aspect is that perceptions of well-being, energy level, or health status may vary from day to day, based on both external and internal factors; thus, the results must not be considered in an absolute way as a trait characteristic, but as a state characteristic and are therefore limited to a specific time window. Another limitation is the reduced number of obese patients. However, we have kept overweight and obese separated since there is not much evidence on the relationship between different weight categories and quality of life, while there are many studies on the obese population. A significant limitation of the study is that HRQoL was evaluated only with KIDSCREEN-52 and no additional factors have been considered, such as socioeconomic status, migration background, and lifestyle habits. Finally, since the questionnaires were completed during a school class, it is possible that the school classroom environment may have biased the students’ responses.

## 5. Conclusions

In conclusion, this study suggests that there is an association between weight status categories and some dimensions of HRQoL and that this association is more pronounced in girls than in boys. Additionally, it is interesting that BMI categories correlate with the psychological perception of well-being in female adolescents, while in boys they affect physical perception, suggesting the importance of implementing obesity prevention strategies that take into account not only metabolic parameters, but also psychological dimensions. Another very important result, in our opinion, is that underweight girls showed a higher score in physical well-being, mood, and self-perception dimensions, pointing out how this altered body satisfaction, in the long term, can contribute to psychological distress potentially leading to eating disorders. These findings, although obtained in a restricted study group of North Italy, are in line with the position statement of the Italian Society for Pediatric Endocrinology and Diabetology and the Italian Society of Pediatrics Against Obesity, suggesting that in the prevention policy perspectives, it is also important to identify all the components of well-being that need to be improved through focused and personalized healthy programs [38].

## Figures and Tables

**Table 1:** Demographic characteristics and BMI classification of the study population, total and by sex.

Variables	Total (*n* = 1707)	Boys (*n* = 828)	Girls (*n* = 879)	*p* Value
Age, (years)	12.5 ± 1.1	12.5 ± 1.1	12.5 ± 1.1	0.420
Underweight	152 (9)	69 (8)	83 (10)	0.01
Normal weight	1310 (77)	621 (75)	689 (78)	
Overweight	194 (11)	105 (13)	89 (10)	
Obesity	51 (3)	33 (4)	18 (2)	

Data are expressed as mean ± standard deviation (SD) or number (%). BMI: body mass index, ns: not significant. For BMI categories, *p* refers to χ^2^ test for trend.

**Table 2:** Sex differences in Kidscreen-52 domains.

Variables	Boys (*n* = 828)	Girls (*n* = 879)	*p*-Value
Physical well-being	50 ± 9 48 ± 9 <0.001	48 ± 9 <0.001	<0.001
Psychological well-being	47 ± 10	47 ± 10	0.549
Mood/Emotion	48 ± 9	46 ± 9	<0.05
Self-perception	52 ± 10	51 ± 11	<0.01
Autonomy	46 ± 9	44 ± 9	<0.001
Parent relationship	50 ± 10	50 ± 11	0.256
Financial resources	48 ± 10	50 ± 10	<0.001
Peers	50 ± 10	50 ± 9	0.417
School environment	48 ± 9	50 ± 8	<0.001
Social acceptance (Bullying)	48 ± 10	49 ± 11	0.164

Data given as mean ± SD. Data on the KIDSCREEN-52 dimension are calculated according to KIDSCREEN Group [15,16]. *p*-values were calculated via Student’s unpaired *t*-test.

**Table 3:** BMI categories according to KIDSCREEN-52 dimensions in males and females.

	Physical Well-Being	Psychological Well-Being	Mood Emotion	Self-Perception	Autonomy	Parent Relationship	Financial Resources	Peers	School Environment	Bullying
Female										
Underweight 83 (9)	50.77 ± 10.39 ^e^*	47.93 ± 12.14	47.86 ± 9.86 ^e^*	53.56 ± 11.36 ^e^*	44.02 ± 10.69 ^d#^	50.10 ± 10.82	48.77 ± 11.38	50.98 ± 12.01	51.56 ± 9.21	49.69 ± 9.73
Normal weight 689 (79)	48.52 ± 8.41	47.67 ± 10.16	46.85 ± 9.28	51.06 ± 11.31 ^b§^	44.06 ± 9.22	50.46 ± 10.78	50.12 ± 9.73	50.06 ± 8.75	50.27 ± 8.2	49.29 ± 10.51
Overweight 89 (10)	47.08 ± 8.54	44.98 ± 10.77	44.4 ± 8.94	45.99 ± 10.11	42.89 ± 9.95	48.83 ± 10.78	47.31 ± 10.05	47.7 ± 10.21	48.87 ± 8.83	46.26 ± 11.43
Obesity 18 (2)	43.49 ± 8.62	44.73 ± 10.42	40.66 ± 10.97	43.08 ± 9.48 ^c^*	42.46 ± 8.47	48.73 ± 11.99	46.65 ± 10.87	48.8 ± 10.57	48.77 ± 11.06	42.67 ± 13.21
*p*	<0.05	0.085	<0.05	<0.001	0.647	0.538	<0.05	0.087	0.177	<0.05
Male										
Underweight 69 (8)	50.91 ± 8.57 ^e^*	46.96 ± 10.23	46.37 ± 8.65	53.19 ± 9.28 ^e^*	46.38 ± 10.51	49 ± 10.35	48.60 ± 9.66	50.69 ± 10.47	48.49 ± 10.01	47.08 ± 10.54
Normal weight 621 (75)	50.42 ± 9.08 ^b^*	42.22 ± 9.94	47.79 ± 8.69	52.25 ± 9.70	46.04 ± 8.78	49.92 ± 9.36	48.23 ± 9.48	49.68 ± 9.62	47.91 ± 8.55	48.56 ± 10.32
Overweight 105 (13)	47.29 ± 8.34	47.64 ± 9.59	47 ± 8.96	50.89 ± 9.88	45.92 ± 9.12	49.74 ± 9.42	47.63 ± 9.17	48.56 ± 11.48	48.59 ± 8.22	47.41 ± 11.06
Obesity 33 (4)	45.46 ± 7.72 ^c^*	42.61 ± 10.22	45.24 ± 11.32	46.95 ± 10.95 ^c^*	42.83 ± 9.43	46 ± 10.61	44.96 ± 10.58	46.68 ± 11.18	45.36 ± 7.9	45.69 ± 10.45
*p*	<0.001	0.068	0.235	<0.05	0.243	0.127	0.245	0.195	0.279	0.256

Data given as mean ± SD or number (%). ns: not significant. Data on the KIDSCREEN-52 dimension are calculated according to KIDSCREEN Group [15,16]. Scheffe post hoc test: ^b^: normal weight vs. overweight; ^c^: normal weight vs. obesity; ^d^: underweight vs. overweight, ^e^: underweight vs. obesity. * *p* < 0.05; ^§^ *p* < 0.01; ^#^ *p* < 0.001.

## Data Availability

The datasets used and/or analyzed during the current study are available from the corresponding author on reasonable request.

## References

[1] Patton G.C., Coffey C., Cappa C., Currie D., Riley L., Gore F., Degenhardt L., Richardson D., Astone N., Sangowawa A.O. (2012). Health of the world’s adolescents: A synthesis of internationally comparable data. Lancet.

[2] Meade T., Dowswell E. (2016). Adolescents′ health-related quality of life (HRQoL) changes over time: A three year longitudinal study. Health Qual. Life Outcomes.

[3] Mak K.K., Tan S.H. (2012). Underweight problems in Asian children and adolescents. Eur. J. Pediatr..

[4] Pu C., Chou Y.J. (2010). Health ratings for underweight, overweight and obese adolescents: Disparities between adolescent’s own report and the parent’s report. Asia Pac. J. Nutr..

[5] World Health Organization (2011). WHO Global Strategy of on Diet, Physical Activity and Health.

[6] Harton A., Myszkowska-Ryciak J., Laskowski W., Gajewska D. (2019). Prevalence of overweight and obesity among adolescents in Poland. J. Health Inequal..

[7] Tragomalou A., Moschonis G., Kassari P., Papageorgiou I., Genitsaridi S.M., Karampatsou S., Manios Y., Charmandari E. (2020). A National e-Health Program for the Prevention and Management of Overweight and Obesity in Childhood and Adolescence in Greece. Nutrients.

[8] Griffiths L.J., Parsons T.J., Hill A.J. (2010). Self-esteem and quality of life in obese children and adolescents: A systematic review. Int. J. Pediatr. Obes..

[9] Viner R.M., Haines M.M., Taylor S.J., Head J., Booy R., Stansfeld S. (2006). Body mass, weight control behaviours, weight perception and emotional wellbeing in a multi-ethnic sample of early adolescents. Int. J. Obes..

[10] Trivellini G., Doveri C., Mastorci F., Bastiani L., Cappa C., Vassalle C., Pingitore A. (2018). Innovative web-based tool for promoting well-being among healthy adolescents: An implementation protocol. J. Transl. Sci..

[11] Mastorci F., Bastiani L., Trivellini G., Doveri C., Vassalle C., Pingitore A. (2020). A new integrated approach for adolescent health and well-being: The AVATAR project. Health Qual. Life Outcomes.

[12] Mastorci F., Bastiani L., Doveri C., Trivellini G., Casu A., Vassalle C., Pingitore A. (2020). Adolescent Health: A Framework for Developing an Innovative Personalized Well-Being Index. Front Pediatr..

[13] de Onis M., Onyango A.W., Borghi E., Siyam A., Nishida C., Siekmann J. (2007). Development of a WHO growth reference for school-aged children and adolescents. Bull World Health Organ..

[14] Cole T.J., Flegal K.M., Nicholls D., Jackson A.A. (2007). Body mass index cut offs to defi ne thinness in children and adolescents: International survey. BMJ.

[15] Ravens-Sieberer U., Gosch A., Rajmil L., Erhart M., Bruil J., Duer W., Auquier P., Power M., Abel T., Czemy L. (2005). KIDSCREEN-52 quality-of-life measure for children and adolescents. Expert Rev. Pharm. Outcomes Res..

[16] The KIDSCREEN Group Europe (2006). The KIDSCREEN Questionnaires—Quality of Life Questionnaires for Children and Adolescents.

[17] Berra S., Ravens-Sieberer U., Erhart M., Tebé C., Bisegger C., Duer W., von Rueden U., Herdman M., Alonso J., Rajmil L. (2007). European KIDSCREEN group. Methods and representativeness of a European survey in children and adolescents: The KIDSCREEN study. BMC Public Health.

[18] Freire T., Ferreira G. (2018). Health-related quality of life of adolescents: Relations with positive and negative psychological dimensions. Int. J. Adolesc. Youth.

[19] Bergman M.M., Scott J. (2001). Young adolescents’ wellbeing and health-risk behaviours: Sex and socio-economic differences. J. Adolesc..

[20] Bisegger C., Cloetta B., Von R.U., Abel T., Ravens-Sieberer U. (2005). Health-related quality of life: Sex differences in childhood and adolescence. Soz Praventivmed.

[21] Michel G., Bisegger C., Fuhr D.C., Abel T. (2009). Age and sex differences in health-related quality of life of children and adolescents in Europe: A multilevel analysis. Qual. Life Res..

[22] Sundar T.K.B., Riiser K., Småstuen M.C., Opheim R., Løndal K., Glavin K., Helseth S. (2020). Health-related quality of life among 13–14 year old adolescents with overweight—A mixed methods approach. Health Qual. Life Outcomes.

[23] Hankin B.L., Abramson L.Y., Moffitt T.E., Silva P.A., McGee R., Angell K.E. (1998). Development of depression from preadolescence to young adulthood: Emerging gender differences in a 10-year longitudinal study. J. Abnorm. Psychol..

[24] Weinstein S.M., Mermelstein R.J., Hankin B.L., Hedeker D., Flay B.R. (2007). Longitudinal Patterns of Daily Affect and Global Mood During Adolescence. J. Res. Adolesc..

[25] Mason A., Rantanen A., Kivim H., Koivisto A., Joronen K. (2016). Family factors and health behaviour of thin adolescent boys and girls. J. Adv. Nurs..

[26] Fallon E.M., Tanofsky-Kraff M., Norman A.C., McDuffie J.R., Taylor E.D., Cohen M.L., Young-Hyman D., Keil M., Kolotkin R.L., Yanovski J.A. (2005). Health-related quality of life in overweight and nonoverweight black and white adolescents. J. Pediatr..

[27] Wawrzyniak A., Myszkowska-Ryciak J., Harton A., Lange E., Laskowski W., Hamulka J., Gajewska D. (2020). Dissatisfaction with Body Weight among Polish Adolescents Is Related to Unhealthy Dietary Behaviors. Nutrients.

[28] Groesz L.M., Levine M.P., Murnen S.K. (2002). The effect of experimental presentation of thin media images on body satisfaction: A meta-analytic review. Int. J. Eat. Disord..

[29] Voelker D.K., Reel J.J., Greenleaf C. (2015). Weight status and body image perceptions in adolescents: Current perspectives. Adolesc. Health Med. Ther..

[30] Bonsergent E., Benie-Bi J., Baumann C., Agrinier N., Tessier S., Thilly N., Briançon S. (2012). Effect of sex on the association between weight status and health-related quality of life in adolescents. BMC Public Health.

[31] Swallen K.C., Reither E.N., Haas S.A., Meier A.M. (2005). Overweight, obesity, and health-related quality of life among adolescents: The National Longitudinal Study of Adolescent Health. Pediatrics.

[32] Strauss R.S. (2000). Childhood obesity and self-esteem. Pediatrics.

[33] Wake M., Salmon L., Waters E., Wright M., Hesketh K. (2002). Parent reported health status of over-weight and obese Australian primary school children: A cross-sectional population survey. Int. J. Obes..

[34] Killedar A., Lung T., Petrou S., Teixeira-Pinto A., Tan E.J., Hayes A. (2020). Weight status and health-related quality of life during childhood and adolescence: Effects of age and socioeconomic position. Int. J. Obes..

[35] Puhl R.M., King K.M. (2013). Weight discrimination and bullying. Best Pract. Res. Clin. Endocrinol. Metab..

[36] Gong Z., Han Z., Zhang H., Zhang G. (2020). Weight Status and School Bullying Experiences in Urban China: The Difference between Boys and Girls. J. Interpers. Violence.

[37] Buckland G., Bach A., Serra-Majem L. (2008). Obesity and the Mediterranean diet. A systematic review of observational and intervention studies. Obes. Rev..

[38] Valerio G., Maffeis C., Saggese G., Ambruzzi M.A., Balsamo A., Bellone S., Bergamini M., Bernasconi S., Bona G., Calcaterra V. (2018). Diagnosis, treatment and prevention of pediatric obesity: Consensus position statement of the Italian Society for Pediatric Endocrinology and Diabetology and the Italian Society of Pediatrics. Ital. J. Pediatr..

